# Supplementary material for: A Fucan Sulfate with Pentasaccharide Repeating Units from the Sea Cucumber Holothuria floridana and Its Anticoagulant Activity
Source: Mar Drugs. 2022 Jun 3;20(6):377. doi: 10.3390/md20060377 (PMC9230062; doi:10.3390/md20060377)
Supplement: Supplementary file 1 [file marinedrugs-20-00377-s001.zip › marinedrugs-1729417-supplementary.pdf]

A fucan sulfate with pentasaccharide repeating units from the sea cucumber *Holothuria floridana* and its anticoagulant activity

Zimo Ning [1], Pin Wang [1], Zhichuang Zuo [1], Xuelin Tao [1], Li Gao [1], Chen Xu [1], Zhiyue Wang [1], Bin Wu[1,*], Na Gao [1,*], Jinhua Zhao [1]

[1] School of Pharmaceutical Sciences, South-Central University for Nationalities, Wuhan, 430074, China; zimo_ning1997@163.com (Z. N.); wangpin1994@163.com (P. W.); 18271682301@163.com (Z. Z.); txl6361@163.com (X. T.); gl15272975735@163.com (L. G.); 15171981226@163.com (C. X.); Wangzhiyue0719@163.com (Z. W.); 2015084@mail.scuec.edu.cn (B. W.); gn2008.happy@163.com (N. G.); zhaojinhua@mail.scue.edu.cn (J. Z)

***** Correspondence: gn2008.happy@163.com (N. G.); 2015084@mail.scuec.edu.cn (B.W.)

Contents

Figure S1. The calibration curve for sulfate standards (A) and sulfate elution profiles of dHfFS-2 obtained by ion chromatography (B).

Figure S2. Overlapped spectra of ^{1}H-^{1}H COSY (gray), TOCSY (red) and ROESY (green) of dHfFS-1.

Figure S3. ^{1}H-^{1}H COSY (A) and ^{1}H-^{13}C HSQC (B) spectra of F1.

Figure S4. Overlapped spectra of ^{1}H-^{1}H COSY (gray), TOCSY (red) and ROESY (blue) of F2.

Figure S5. SAX chromatography of F3 and ^{1}H NMR spectra of F3, F3-a, F3-b and F3-c.

Figure S6. ^{1}H-^{13}C HSQC (gray) and HMBC (red) spectra of F3-a.

Figure S7. Overlapped spectra of ^{1}H-^{1}H COSY (gray), TOCSY (red) and ROESY (green) of F3-b.

Figure S8. ^{1}H-^{13}C HSQC and HMBC spectra of F4.

Figure S9. Overlapped spectra of ^{1}H-^{1}H COSY (gray), TOCSY (red) and ROESY (green) of F5.

Table S1. ^{1}H and ^{13}C chemical shifts of the oligosaccharide F1.

Table S2. ESI-MS data assignments of fucooligosaccharides.

Table S3. ^{1}H and ^{13}C chemical shifts of the fucooligosaccharide **F3-a** and **F3-b.**

NMR acquisition parameters.

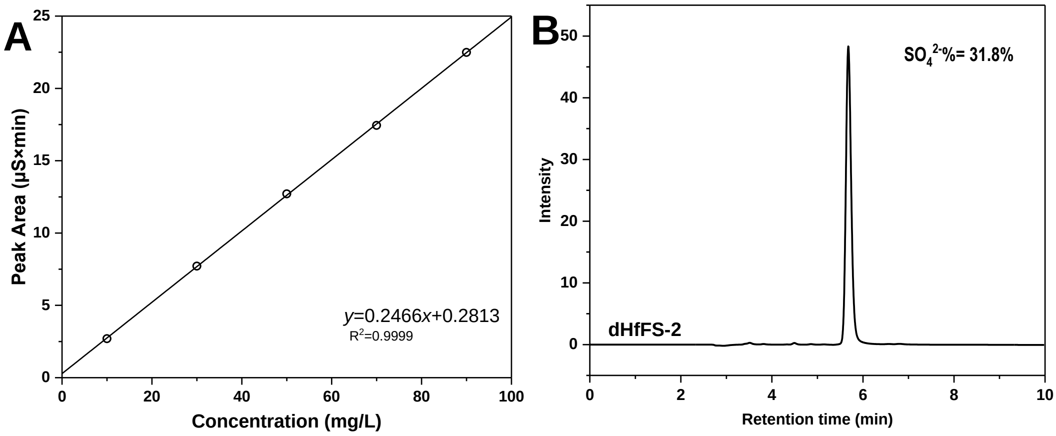

Figure S1. The calibration curve for sulfate standards (A) and sulfate elution profiles of dHfFS-2 obtained by ion chromatography (B).

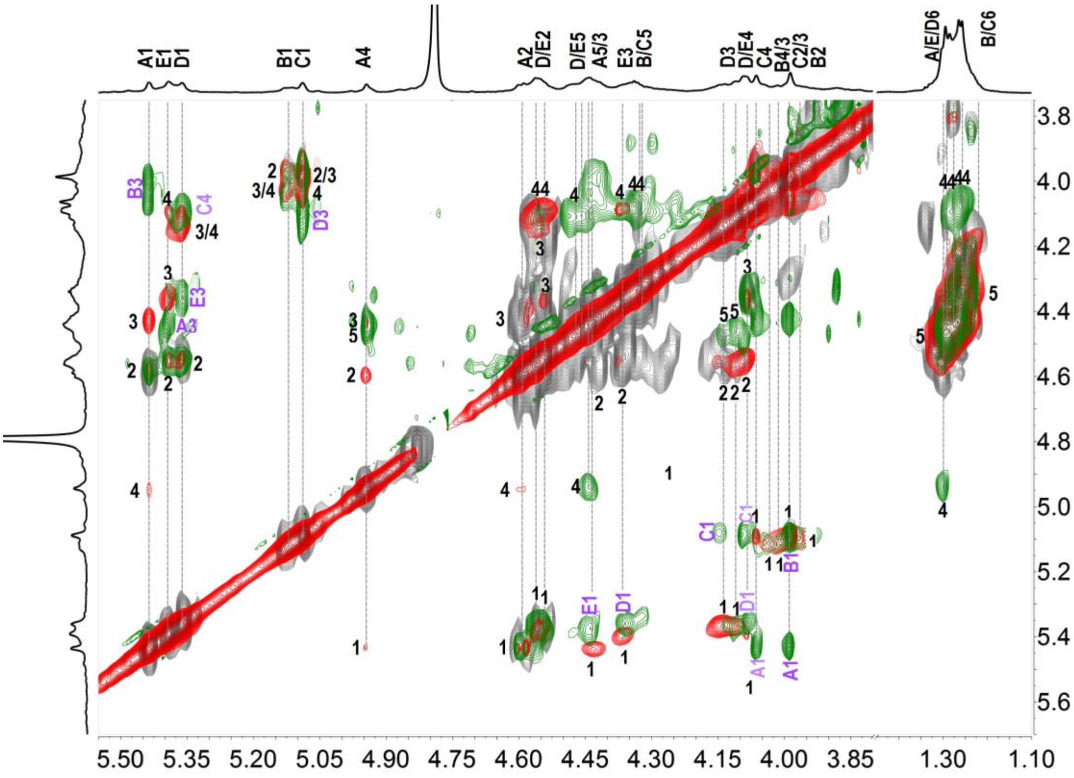

Figure S2. Overlapped spectra of ^{1}H-^{1}H COSY (gray), TOCSY (red) and ROESY (green) of dHfFS-1.

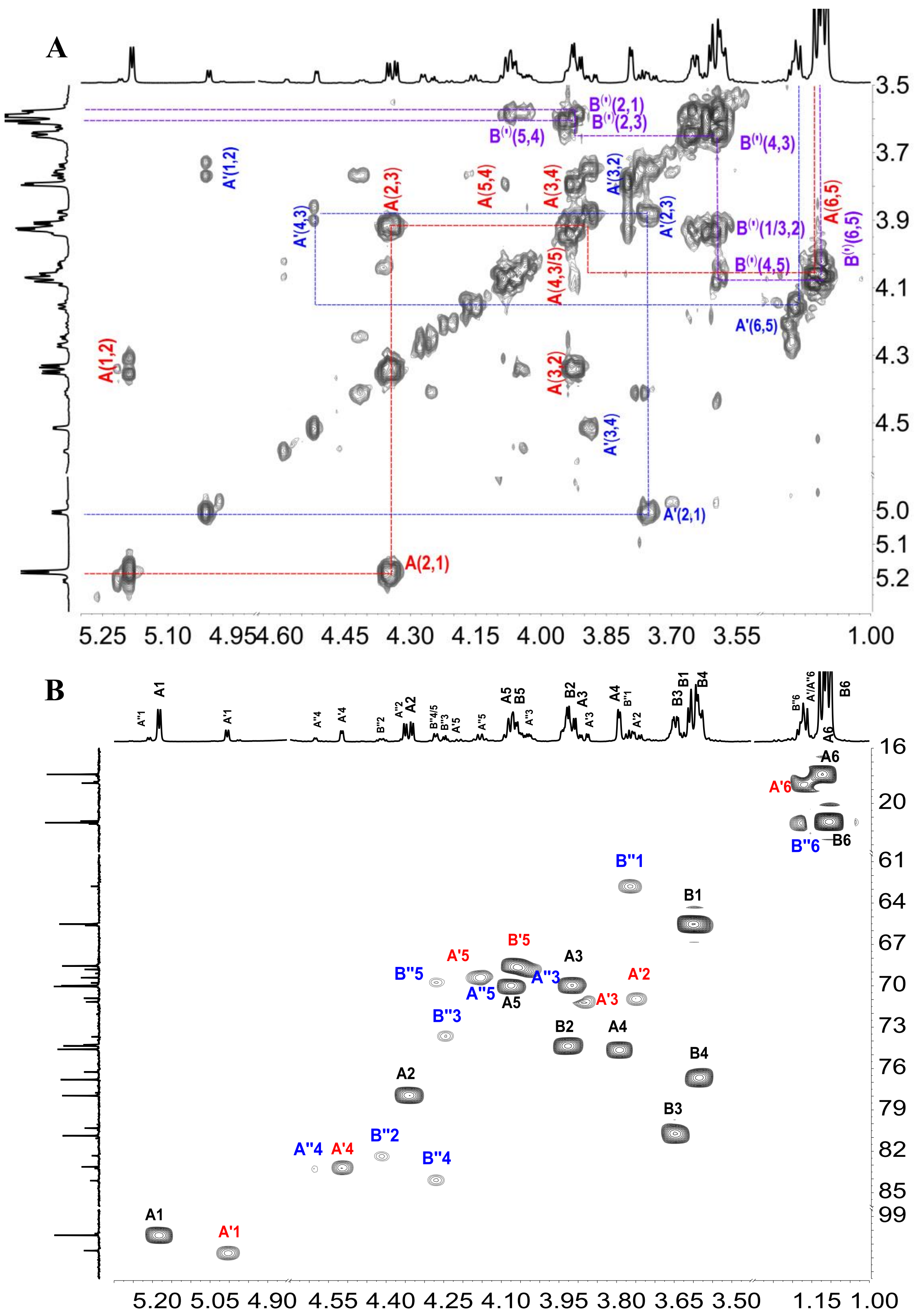

Figure S3. ^{1}H-^{1}H COSY (A) and ^{1}H-^{13}C HSQC (B) spectra of F1.

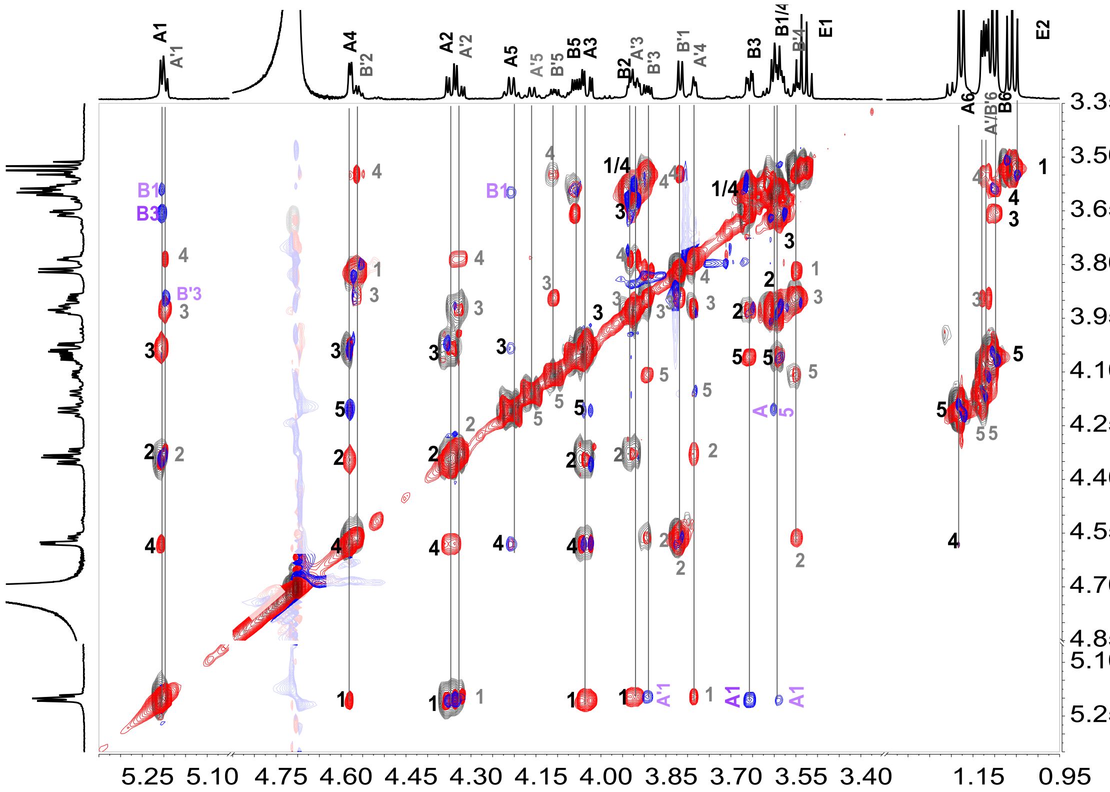

Figure S4. Overlapped spectra of ^{1}H-^{1}H COSY (gray), TOCSY (red) and ROESY (blue) of F2.

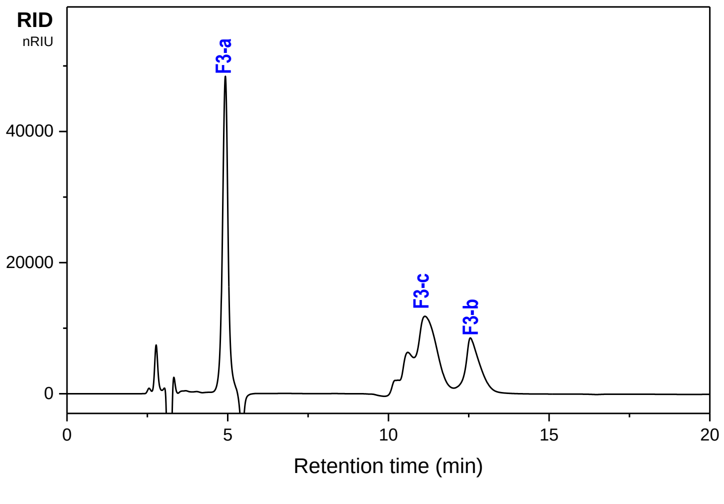

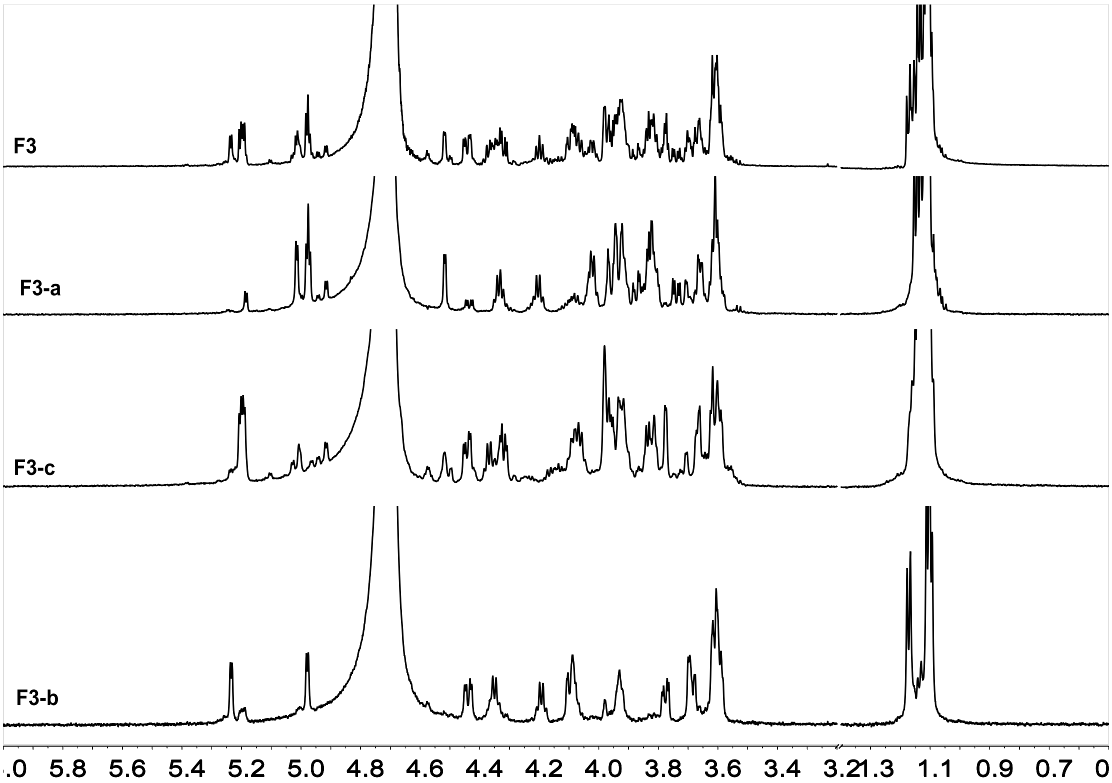

Figure S5. SAX chromatography of F3 and ^{1}H NMR spectra of F3, F3-a, F3-b and F3-c.

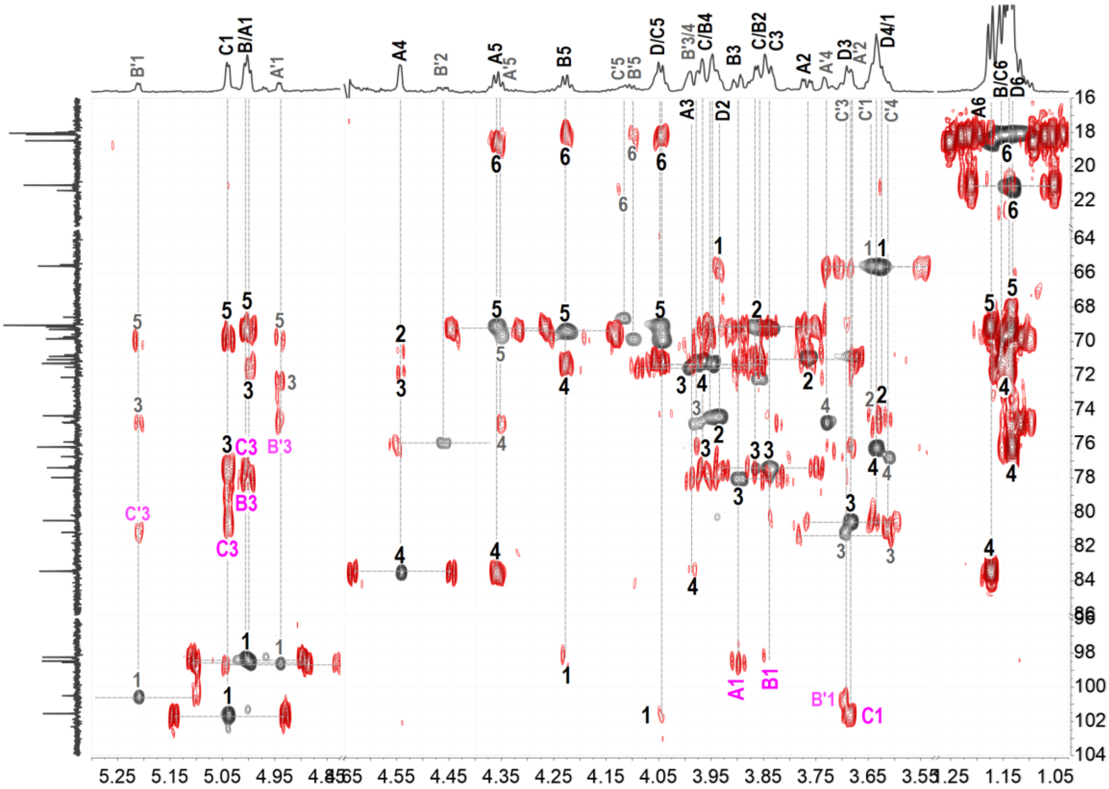

Figure S6. ^{1}H-^{13}C HSQC (gray) and HMBC (red) spectra of F3-a.

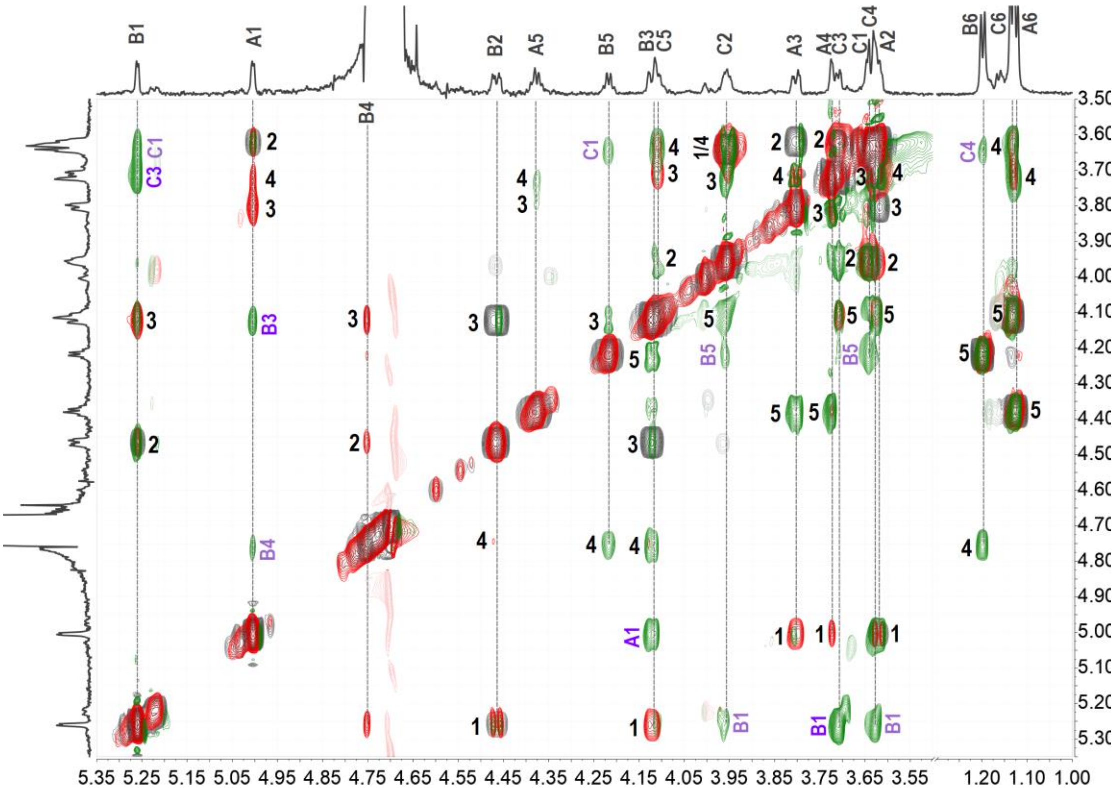

Figure S7. Overlapped spectra of ^{1}H-^{1}H COSY (gray), TOCSY (red) and ROESY (green) of F3-b.

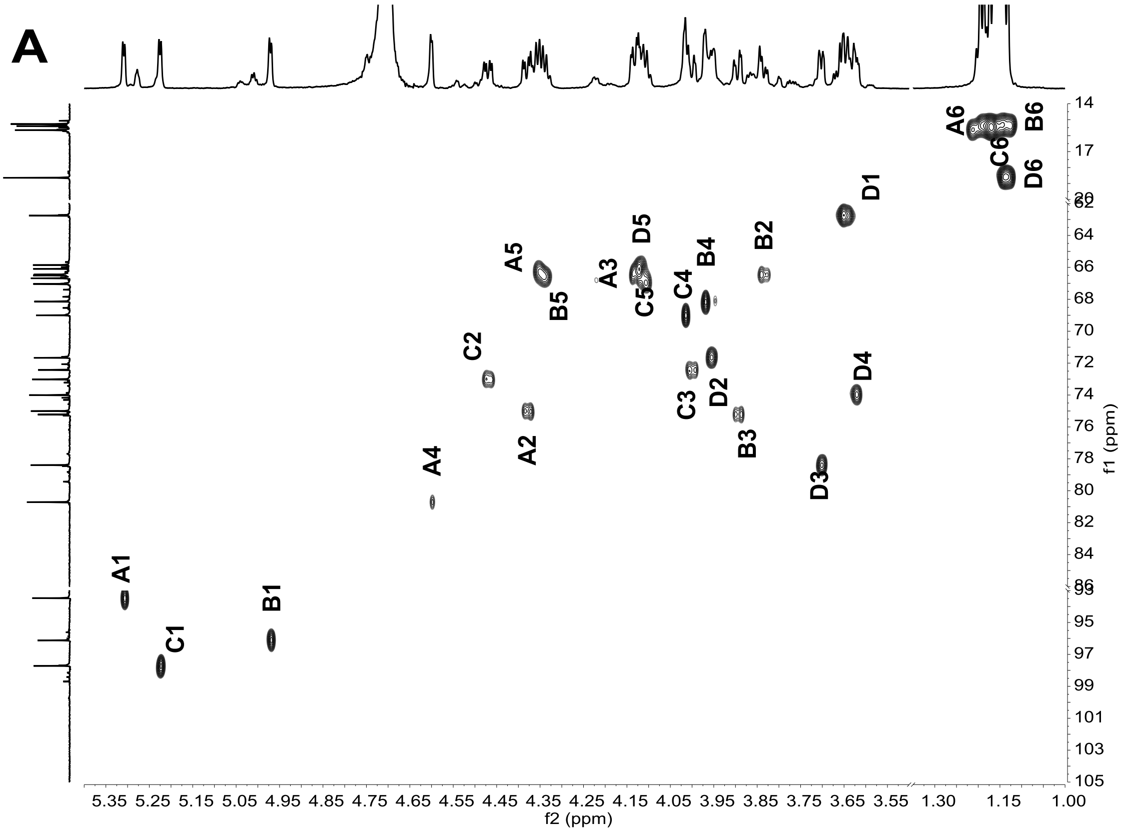

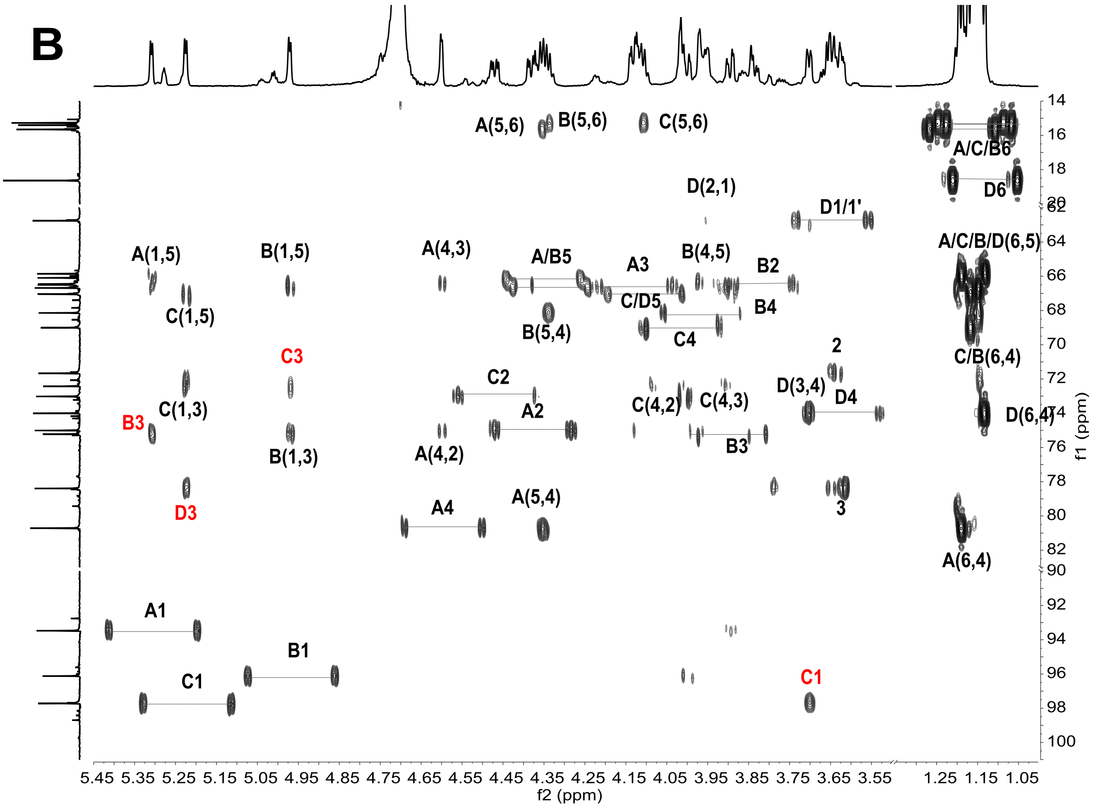

Figure S8. ^{1}H-^{13}C HSQC and HMBC spectra of F4.

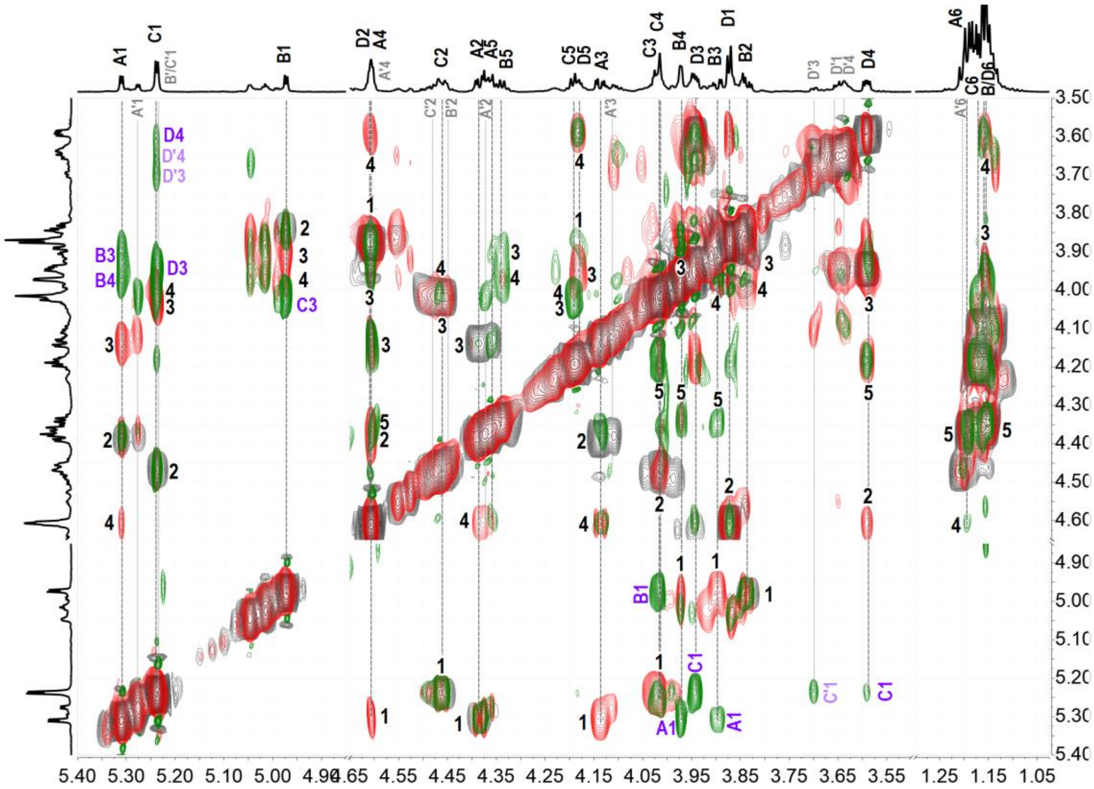

Figure S9. Overlapped spectra of ^{1}H-^{1}H COSY (gray), TOCSY (red) and ROESY (green) of F5.

Table S1 ^{1}H and ^{13}C chemical shifts of the oligosaccharide F1

<table>
<tr><th rowspan="2">Fraction</th><th rowspan="2">Residue</th><th rowspan="2"></th><th colspan="6">Chemical shift (ppm)</th></tr>
<tr><th>1</th><th>2</th><th>3</th><th>4</th><th>5</th><th>6</th></tr>
<tr><td rowspan="5">F1-a</td><td rowspan="2">A: L-Fuc$_{2S}$-α(1</td><td>H</td><td>5.183 ($J_{1,2}$=4.02)</td><td>4.342</td><td>3.918</td><td>3.793</td><td>4.079</td><td>1.125</td></tr>
<tr><td>C</td><td>100.37</td><td>77.98</td><td>70.00</td><td>74.61</td><td>70.05</td><td>17.90</td></tr>
<tr><td rowspan="3">B: -3)-L-Fuc-ol</td><td rowspan="2">H</td><td>3.627 ($J_{1,1'}$=11.58)</td><td>3.935</td><td>3.647</td><td>3.591</td><td>4.066</td><td>1.105</td></tr>
<tr><td>3.597 ($J_{1,2}$=4.74)</td><td></td><td></td><td></td><td></td><td></td></tr>
<tr><td>C</td><td>65.53</td><td>74.36</td><td>80.89</td><td>76.81</td><td>68.56</td><td>21.40</td></tr>
<tr><td rowspan="5">F1-b</td><td rowspan="2">A': L-Fuc$_{4S}$-α(1-</td><td>H</td><td>5.006 ($J_{1,2}$=4.20)</td><td>3.747</td><td>3.884</td><td>4.515</td><td>4.156</td><td>1.171</td></tr>
<tr><td>C</td><td>101.49</td><td>70.91</td><td>71.17</td><td>83.14</td><td>69.42</td><td>18.53</td></tr>
<tr><td rowspan="3">B': -3)-L-Fuc-ol</td><td rowspan="2">H</td><td>3.623 ($J_{1,1'}$=11.58)</td><td>3.926</td><td>3.664</td><td>3.582</td><td>4.032</td><td>1.112</td></tr>
<tr><td>3.597 ($J_{1,2}$=4.74)</td><td></td><td></td><td></td><td></td><td></td></tr>
<tr><td>C</td><td>65.63</td><td>74.26</td><td>80.33</td><td>76.26</td><td>68.82</td><td>21.28</td></tr>
<tr><td rowspan="5">F1-c</td><td rowspan="2">A'': L-F$_{2S4S}$-α(1-</td><td>H</td><td>5.209 ($J_{1,2}$=4.08)</td><td>4.350</td><td>4.032</td><td>4.584</td><td>4.211</td><td>1.184</td></tr>
<tr><td>C</td><td>100.25</td><td>77.77</td><td>69.11</td><td>83.25</td><td>69.80</td><td>18.43</td></tr>
<tr><td rowspan="3">B'': -3)-L-Fuc-ol</td><td rowspan="2">H</td><td>3.623 ($J_{1,1'}$=11.58)</td><td>3.926</td><td>3.664</td><td>3.582</td><td>4.032</td><td>1.112</td></tr>
<tr><td>3.597 ($J_{1,2}$=4.74)</td><td></td><td></td><td></td><td></td><td></td></tr>
<tr><td>C</td><td>65.51</td><td>74.31</td><td>81.08</td><td>76.81</td><td>68.56</td><td>21.28</td></tr>
<tr><td rowspan="2">F1-d</td><td rowspan="2">rF: L-F$_{2S4S}$-ol</td><td>H</td><td>3.764 ($J_{1,2}$=4.56)</td><td>4.412</td><td>4.248</td><td>4.273</td><td>4.269</td><td>1.105</td></tr>
<tr><td>C</td><td>62.79</td><td>82.33</td><td>73.70</td><td>84.14</td><td>69.80</td><td>21.49</td></tr>
</table>

Table S2. ESI-MS data assignments of fucooligosaccharides

Sample	Actual Peak	Charge	Negative Ion		Predicted Peak
			Structure	Formula	
**F1**	391.0932	1-	$[M-Na]^-$	$C_{12}H_{23}O_{12}S^-$	391.0916
**F2**	493.0325	1-	$[M-Na]^-$	$C_{12}H_{22}NaO_{15}S_2^-$	493.0303
	471.0502	1-	$[M-2Na+H]^-$	$C_{12}H_{23}O_{15}S_2^-$	471.0484
**F3-a**	683.2080	1-	$[M-Na]^-$	$C_{24}H_{43}O_{20}S^-$	683.2074
**F3-b**	639.0886	1-	$[M-Na]^-$	$C_{18}H_{32}NaO_{19}S_2^-$	639.0882
	537.1501	1-	$[M-SO_3-2Na+H]^-$	$C_{18}H_{33}O_{16}S^-$	537.1495
**F4**	887.0847	1-	$[M-Na]^-$	$C_{24}H_{41}Na_2O_{26}S_3^-$	887.0849
	865.1022	1-	$[M-2Na+H]^-$	$C_{24}H_{42}NaO_{26}S_3^-$	865.1030
	785.1461	1-	$[M-SO_3-2Na+H]^-$	$C_{24}H_{42}NaO_{23}S_2^-$	785.1461
	763.1637	1-	$[M-SO_3-3Na+2H]^-$	$C_{24}H_{43}O_{23}S_2^-$	763.1462
**F5**	1011.1595	1-	$[M-H]^-$	$C_{24}H_{39}Na_4O_{29}S_4^-$	1011.0056
	989.0233	1-	$[M-Na]^-$	$C_{24}H_{40}Na_3O_{29}S_4^-$	989.0237
	909.2215	1-	$[M-SO_3-Na]^-$	$C_{24}H_{40}Na_3O_{26}S_3^-$	909.0669
	887.0843	1-	$[M-SO_3-2Na+H]^-$	$C_{24}H_{41}Na_2O_{26}S_3^-$	887.0849
	865.1022	1-	$[M-SO_3-3Na+2H]^-$	$C_{24}H_{42}NaO_{29}S_3^-$	865.1030

Table S3. ^{1}H and ^{13}C chemical shifts of the fucooligosaccharide **F3-a** and **F3-b**

Fraction	Residue		chemical shift (ppm)					
			1	2	3	4	5	6
**F3-a-I**	**A: L-Fuc$_{4S}$-α(1-**	H	4.999 ($J_{1,2}$=3.60)	3.766	3.983	4.543	4.360	1.174
		C	98.48	70.88	71.30	83.44	69.06	18.48
	**B: -3)-L-Fuc-α(1-**	H	4.005 ($J_{1,2}$=4.16)	3.842	3.899	3.948	4.228	1.139
		C	98.25	69.16	77.96	71.28	69.30	18.12
	**C: -3)-L-Fuc-α(1-**	H	5.039 ($J_{1,2}$=3.36)	3.854	3.836	3.966	4.045	1.152
		C	101.54	69.06	77.39	71.06	69.85	18.12
	**D: -3)-L-Fuc-ol**	H	3.630	3.938	3.683	3.636	4.048	1.133
		C	65.60	74.34	80.48	76.18	69.07	21.09
**F3-a-II**	**A': L-Fuc-α(1-**	H	4.941 ($J_{1,2}$=3.60)	3.680	3.858	3.732	4.352	1.121
		C	98.55	70.79	72.10	74.73	69.73	18.00
	**B': -3)-L-Fuc$_{2S}$-α(1-**	H	5.210 ($J_{1,2}$=3.68)	4.462	3.983	3.993	4.101	1.165
		C	100.05	75.89	74.76	71.51	69.82	18.05
	**C': -3)-L-Fuc-ol**	H	3.643 ($J_{1,2}$=3.36)	3.950	3.696	3.613	4.109	1.148
		C	65.52	74.40	81.17	76.74	68.58	21.40
**F3-b**	**A: L-Fuc-α(1-**	H	5.003 ($J_{1,2}$=4.24)	3.622	3.803	3.723	4.376	1.125
		C	100.66	71.05	72.44	74.72	70.05	18.12
	**B: -3)-L-Fuc$_{2S4S}$-α(1-**	H	5.260 ($J_{1,2}$=3.68)	4.465	4.122	4.750	4.217	1.198
		C	100.47	75.84	75.08	81.91	69.70	18.41
	**C: -3)-L-Fuc-ol**	H	3.363	3.956	3.709	3.626	4.110	1.133
		C	100.89	74.33	81.32	76.86	68.69	21.39

NMR acquisition parameters for F1 and F2 on 600 MHz NMR spectrometer

^{1}H NMR: Number of scans (NS), 16; Spectral width in hertz (SWH), 12019.2 Hz; Acquisition time (AQ), 2.7263; Receiver gain (RG), 30.1; Temperature (TE), 295.0 K; Relaxation delay (D1), 1.0 sec; Pulse width (P1), 11.5 μsec; Size of real spectrum (SI), 65536.

^{13}C NMR: NS, 300; SWH, 36231.9 Hz; AQ, 0.9044; RG, 190.7; TE, 295.0 K; D1, 2.0 sec; P1, 10.00 μsec; SI, 32768.

^{1}H-^{1}H COSY: NS, 4; SWH, 4807.7, 4803.1 Hz; AQ, 0.2130; RG, 190.7; TE, 295.0 K; D1, 2.0 sec; P1, 11.5 μsec; SI, 1024.

^{1}H-^{1}H TOCSY: NS, 8; SWH, 4807.7, 4803.1 Hz; AQ, 0.2130; RG, 190.7; TE, 295.0 K; D1, 2.0 sec; P1, 11.5 μsec; SI, 1024.

^{1}H-^{1}H ROESY: NS, 8; SWH, 4807.7, 4803.1 Hz; AQ, 0.2130; RG, 190.7; TE, 295.0 K; D1, 2.0 sec; P1, 11.5 μsec; SI, 1024.

^{1}H-^{13}C HSQC: NS, 2; SWH, 4807.7, 22624.4 Hz; AQ, 0.1065; RG, 190.7; TE, 295.0 K; D1, 1.5 sec; P1, 11.5 μsec.

^{1}H-^{13}C HMBC: NS, 10; SWH, 4807.7, 25641.0 Hz; AQ, 0.4160; RG, 190.7; TE, 295.0 K; D1, 1.5 sec; P1, 11.5 μsec.

NMR acquisition parameters for dHfFS-1, F3-a, F3-b, F4 and F5 on 800 MHz NMR spectrometer

^{1}H NMR: Number of scans (NS), 4; Spectral width in hertz (SWH), 16025.6 Hz; Acquisition time (AQ), 2.0447; Receiver gain (RG), 5.7; Temperature (TE), 298.0 K; Relaxation delay (D1), 2.0 sec; Pulse width (P1), 7.1 μsec; Size of real spectrum (SI), 131072.

^{13}C NMR: NS, 2000; SWH, 48076.9 Hz; AQ, 0.6816; RG, 2050; TE, 298.0 K; D1, 2.0 sec; P1, 12.00 μsec; SI, 32768.

^{1}H-^{1}H COSY: NS, 8; SWH, 8012.8, 8012.8 Hz; AQ, 0.1278; RG, 654.4; TE, 298.0 K; D1, 1.0 sec; P1, 7.38 μsec; SI, 1024.

^{1}H-^{1}H TOCSY: NS, 8; SWH, 8081.9, 8077.5 Hz; AQ, 0.1267; RG, 1440; TE,

298.0 K; D1, 1.5 sec; P1, 7.38 μsec; SI, 1024.

^{1}H-^{1}H ROESY: NS, 8; SWH, 8012.8, 8012.8 Hz; AQ, 0.1278; RG, 757.8; TE, 298.0 K; D1, 1.5 sec; P1, 7.38 μsec; SI, 1024.

^{1}H-^{13}C HSQC: NS, 8; SWH, 8012.8, 20161.3 Hz; AQ, 0.1278; RG, 2050; TE, 298.0 K; D1, 1.5 sec; P1, 7.38 μsec.

^{1}H-^{13}C HMBC: NS, 16; SWH, 8012.8, 34246.6 Hz; AQ, 0.2556; RG, 2050; TE, 298.0 K; D1, 1.5 sec; P1, 7.38 μsec.
